# Longitudinal Analysis of Psychological Distress in Grandparents of Children With Cancer: Results From a Multicenter Cohort Study in Switzerland (GROKids Project)

**DOI:** 10.1002/cam4.71774

**Published:** 2026-04-07

**Authors:** Barbara Gantner, Cristina Priboi, Anica Ilic, Freimut H. Schilling, Ahmed Farrag, Katrin Scheinemann, Marc Ansari, Jeanette Greiner, Nicolas von der Weid, Pierlugi Brazzola, Manuel Diezi, Elena Lemmel, Pauline Holmer, Peter Francis Raguindin, Gisela Michel

**Affiliations:** ^1^ Faculty of Health Sciences and Medicine University of Lucerne Lucerne Switzerland; ^2^ Consultation‐Liaison Psychiatry of the Children’s Hospital of Central Switzerland Lucerne Psychiatric Services Lucerne Switzerland; ^3^ Veterinary Public Health Institute University of Bern Bern Switzerland; ^4^ Institute for Biomedical Ethics University of Basel Basel Switzerland; ^5^ Faculty of Psychology and Educational Sciences University of Geneva Geneva Switzerland; ^6^ Institute of Basic Medical Sciences, Faculty of Medicine University of Oslo Oslo Norway; ^7^ Division of Pediatric Hematology and Oncology, Department of Pediatrics Children’s Hospital of Central Switzerland Lucerne Switzerland; ^8^ Pediatric Oncology Department, South Egypt Cancer Institute Assiut University Assiut Egypt; ^9^ Division of Hematology/Oncology Children’s Hospital of Eastern Switzerland St. Gallen Switzerland; ^10^ CANSEARCH Research Platform for Pediatric Oncology and Hematology, Department of Pediatrics, Gynecology and Obstetrics, Faculty of Medicine University of Geneva Geneva Switzerland; ^11^ Division of Pediatric Oncology and Hematology, Department of Women, Child and Adolescent Geneva Unversity Hospitals Geneva Switzerland; ^12^ Pediatric Oncology‐Hematology, Children’s Hospital Cantonal Hospital Aarau Aarau Switzerland; ^13^ Department of Pediatric Oncology and Hematology University Children’s Hospital Basel Basel Switzerland; ^14^ Emato‐Oncologia Pediatrica, Istituto Pediatrico Della Svizzera Italiana Ospedale Regionale di Bellinzona e Valli Bellinzona Switzerland; ^15^ Pediatric Hematology and Oncology Unit, Department of Pediatrics Lausanne University Hospital Lausanne Switzerland; ^16^ Faculty of Human Sciences, Department of Inclusive Education University of Potsdam Potsdam Germany

**Keywords:** anxiety, childhood cancer, depression, grandparents, psychological distress, somatization

## Abstract

**Objectives:**

This exploratory study assessed psychological distress of grandparents of children recently diagnosed with cancer within 2 years post‐diagnosis. We examined changes over time, identified associated factors, and compared distress levels with a comparison group (grandparents of survivors).

**Methods:**

Grandparents were recruited via parents of children recently diagnosed with cancer (within 3 months). Survivors (3–10 years post‐diagnosis) were asked to provide contact information of their grandparents (comparison group). Grandparents completed surveys at 3 (T1), 6 (T2), 12 (T3), and 24 months (T4) post‐diagnosis. The comparison group completed a questionnaire once. Psychological distress was assessed using the Brief Symptom Inventory‐18, with three subscales (depression, anxiety, somatization), and an overall score (Global Severity Index; GSI). Scores were T‐standardized. We used linear mixed models to compare distress across time points and linear regressions for between‐group differences (grandparents versus comparison group).

**Results:**

Forty‐one grandparents (mean age = 68 years, 59% female) of 20 patients participated. In the comparison group 133 grandparents (mean age = 72 years, 60% female) of 66 survivors (mean years post‐diagnosis = 6.7, SD = 2.3) participated. GSI scores were all below normative values. Increases in GSI were observed from T1 to T2 (24%) and T3 to T4 (26%). Greater distance from the child's hospital was associated with lower GSI (*β*: −13.27, *p* = 0.033) and anxiety scores (*β*: −5.50, *p* = 0.017). Grandfathers reported higher levels of somatization than grandmothers (*β*: 4.00, *p* = 0.071). Older age was associated with lower anxiety (*β*: −9.86, *p* = 0.042).

**Conclusion:**

Although most grandparents reported psychological distress within normative ranges, distress levels increased in some. Targeted interventions might help support grandparents of children with cancer.

**Abbreviations**
BSI: Brief Symptom Inventory 18
CI: Confidence interval
CNS: Central nervous system
GSI: Global Severity Index
RCI: Reliable Change Index
SD: Standard deviation

## 1 Background

A grandchild's cancer diagnosis imposes substantial emotional burden on grandparents [1]. Although providing emotional and practical support to the family [2], grandparents also experience significant distress, often reporting shock, fear, and helplessness [3, 4]. Many describe it as the “worst experience of their lives” [3]. Beyond the grandchild with cancer, they also worry about the child's parents [5, 6] and siblings [7, 8]. This triple burden of concern has a significant impact on their emotional and psychological well‐being [9]. Previous research has shown that grandparents experience fear [3, 6, 10], higher distress and depression [4], lower quality of life [11], and report deterioration in their mental health compared to grandparents whose grandchildren were healthy [11]. Sleep disturbances and increased use of medication have also been reported as common manifestations of stress [11]. In addition, concerns about the grandchild's long‐term psychological outcomes may further intensify their emotional strain [1].

Beyond emotional distress, the cancer diagnosis reshapes family dynamics significantly. Although it often brings families closer together, enhancing cohesion and a shared purpose in dealing with the crisis, it can also introduce stress and strain within family relationships [3, 9]. Grandparents often assume supportive roles, providing emotional and practical assistance not only to the child but also to their adult children who are managing caregiving demands [12]. This role shift can push grandparents deeper into familial responsibilities, potentially affecting their overall quality of life [4, 11]. Although such involvement can bring a sense of fulfillment, the demands of the situation can also lead to feelings of being overwhelmed and underappreciated [3].

Most existing studies on grandparents have focused on contexts other than pediatric cancer such as chronic illnesses or disabilities [2, 8]. Evidence on grandparents' psychological distress immediately following a cancer diagnosis and during the intensive treatment phase is limited. To date, no longitudinal studies have examined trajectories of distress over time, and few studies have investigated potential predictors of grandparents' distress during their grandchild's cancer treatment.

Given the involvement of grandparents, it is important to examine grandparents' mental health when their grandchild is diagnosed with cancer. We aimed to (a) describe the longitudinal changes in psychological distress of grandparents in the first 2 years after their grandchild's cancer diagnosis, (b) identify factors associated with psychological distress, namely, sociodemographic, child‐related, and cancer‐related factors, and (c) compare the psychological distress of grandparents of recently diagnosed children with the psychological distress of a comparison group (grandparents of survivors).

## 2 Methods

This exploratory cohort analysis is based on a multicenter study aiming at identifying the biopsychosocial outcomes of a childhood cancer diagnosis on grandparents (GROKids Project) [13]. The STROBE checklist was used to guide transparent reporting of observational study (see Supporting Information).

### 2.1 Sample and Procedure

#### 2.1.1 Grandparents of Childhood Cancer Patients

We will use the term “grandparents” for grandparents with a grandchild undergoing cancer treatment. Grandparents were eligible for this study if they (a) were fluent in German, French or Italian, and if they had a grandchild who (b) had been diagnosed with cancer within the previous 3 months, (c) had received the diagnosis before 18 years of age, and (d) was undergoing cancer therapy in one of eight Swiss pediatric oncology centers participating in the study. Grandparents of patients who did not receive any treatment (“watch and wait”) or needed palliative treatment were excluded. No child transitioned to palliative care during the study period. More than one grandparent per child was allowed to participate.

Each participating pediatric oncology center identified eligible childhood cancer patients (grandchildren). Parents of eligible grandchildren received an information package from the hospital staff, which contained detailed information about the study, an informed consent form, an invitation for the grandparents to participate, and a form to provide grandparents contact details. Each parent completed a form to allow the grandparents to be contacted. The study team at the University of Lucerne then contacted the grandparents with an information letter. After providing written informed consent, grandparents received a questionnaire to complete either on paper or online at the following timepoints: T1 at 3 months, T2 at 6 months, T3 at 1 year, and T4 at 2 years after the diagnosis of childhood cancer. Reminders were sent after 4 weeks of no response, followed by call reminders. Cards were sent during special holidays to improve cohort retention. A clinical psychologist was available to grandparents for counseling as needed. Enrollment was open from October 2020 to March 2023, follow‐up was continued until December 2024.

#### 2.1.2 Comparison Group (Grandparents of Childhood Cancer Survivors)

As comparisons, we used a sample of grandparents of childhood cancer survivors from the cross‐sectional study in the GROKids Project [14]. Eligibility criteria were identical to those described above, except that the cancer diagnosis occurred 3–10 years before study participation and treatment had been completed at least 2 years prior.

The comparison group was recruited using a structured, multi‐step procedure. First, the participating hospitals identified eligible cases (survivors) within their respective database. Second, adult survivors or the parents of minor (child) survivor were informed about the study by post. Third, families who expressed interest provided grandparents' contact details using a standardized contact form, which was returned to the study team in a prepaid envelope. Fourth, informed consent forms were sent to eligible grandparents. Fifth, upon receipt of written consent, a paper‐based questionnaire was mailed, with the option to complete the survey online. If no response was received, a reminder letter was sent. Data collection was conducted between December 2021 and July 2023.

### 2.2 Ethical Approval

This study was approved by the Ethical Committee of Northwest and Central Switzerland (EKNZ 2020‐01409, 26 August 2020). Written informed consent was obtained from all study participants.

### 2.3 Measurements

#### 2.3.1 Psychological Distress

Psychological distress was measured using the Brief Symptom Inventory‐18 (BSI‐18) [15], which is validated in the Swiss population [16]. The questionnaire consists of three symptom domains: somatization, depression, and anxiety. Each domain contains six items that were answered on a 5‐point Likert scale from 0 (“not at all”) to 4 (“extremely”). Each domain was analyzed individually (sum of the domain items), and in addition, an overall score was calculated [sum of all 18 items, Global Severity Index (GSI)]. The scores were T‐standardized (mean = 50, SD = 10) [17]. Reaching a T‐score ≥ 57 on the GSI or in two out of three domains was defined as clinically significant distress [18]. For missing items (a maximum of 1 item per domain and a maximum of 3 for the GSI), domain scores or GSI were calculated by imputing missing items with the domain average of the remaining items. Otherwise, the domain and/or GSI were considered missing.

#### 2.3.2 Sociodemographic Characteristics

Participants reported sociodemographic characteristics including age, gender (female vs. male), nationality (Swiss vs. non‐Swiss), education [low (compulsory school) vs. middle (vocational training or high school graduation) vs. high (upper secondary or university education)], household income levels (≤ 6000 CHF vs. > 6000 CHF/month), employment status (employed vs. unemployed or retired), and relationship status (in partnership vs. single).

The following data on child‐related characteristics were also self‐reported: the gender of the ill grandchild (female vs. male), the total number of grandchildren (1–2 vs. > 2), kinship (maternal vs. paternal grandparents) and for the grandparents hospital proximity (travel time in hours between the ill grandchild's hospital and grandparents, reclassified to < 0.5 h vs. 0.5–1 h vs. 1–1.5 h vs. more than 1.5 h).

#### 2.3.3 Cancer‐Related Characteristics

The following cancer‐related characteristics were obtained from the medical records: type of childhood cancer (leukemia or lymphoma vs. other types of cancer), age of the ill grandchild (as continuous variable in years) and type of treatment (chemotherapy vs. radiotherapy or surgery or combination).

### 2.4 Data Analysis

All analyses were conducted using Stata version 18.0. All statistical tests were two‐sided *p*‐values were reported accordingly. All results with *p* < 0.10 are discussed, but *p* < 0.05 was considered the cut‐off for statistical significance.

#### 2.4.1 Longitudinal Changes of Psychological Distress of Grandparents (Aim 1)

Linear mixed models using repeated measures approach were used to determine the changes of means across four time points. Random‐intercept models were fitted using T‐scores of GSI, somatization, anxiety, and depression as outcome, and individual as cluster. Time (T1, reference; T2; T3; T4) was included as fixed effect. This model measured the mean change (beta‐coefficient) in outcomes across time. Crude and gender‐adjusted models were fitted. Due to sample size limitation, additional model adjustments were not performed.

The Reliable Change Index (RCI) was calculated for the GSI to determine whether changes from T1 to T2, T2 to T3, and T3 to T4 reflected statistically reliable change or normal measurement variability [19]. The RCI was computed using the GSI Cronbach's alpha and the standard error of the difference between consecutive time points (*T*_*x*_ and *T*_*x* + 1_) [20], and GSI scores were classified as showing an increase, decrease, or no change across time.

#### 2.4.2 Determinants of Psychological Distress (Aim 2)

To identify possible predictors for psychological distress, linear mixed models were used. Univariable regressions were fitted using *T*‐scores of the domains separately: GSI, depression, somatization, and anxiety as outcome. Each regression model included a single predictor variable. The predictors were time‐points and sociodemographic (gender, migration background, education, household income level, employment, and relationship status), child‐related (age, gender, total number of grandchildren, kinship relationship, hospital proximity) and cancer‐related (age at diagnosis, time since diagnosis, type of cancer, cancer therapy) characteristics. A random intercept model was used, with individuals treated as clusters. Due to sample size limitations, only univariable regressions were conducted and interaction terms with time were not included or an additional cluster for family was not fitted.

#### 2.4.3 Comparison of Psychological Distress of Grandparents and the Comparison Group (Aim 3)

Linear regression was used to compare psychological distress of grandparents with the comparison group at each timepoint. Psychological distress (*T*‐scores of GSI, depression, somatization and anxiety) at each timepoint (T1, T2, T3, T4) was the outcome, and group membership (grandparents vs. comparison group) the predictor. Regression models were adjusted for age to account for difference between the group.

## 3 Results

A total of 85 grandparents (grandparents of patients) were approached for the study, of whom 41 grandparents of 20 patients participated (Table 1). Questionnaires were completed by 39 grandparents at T1, 34 at T2, 34 at T3 and 32 at T4, with 27 grandparents participating at four time points (Figure S1). Grandparents were on average 67.7 years old and more grandmothers (58.5%) took part than grandfathers. The majority were not employed or retired (60.5%). The majority of participants were in a partnership (89.5%). The patients (63.1% females) were on average 6.1 years old at the time of diagnosis. All grandparents had more than one grandchild. Most patients had leukemia (52.6%) and had been treated with a combination of chemotherapies, surgery, or radiotherapy (68.4%).

**TABLE 1:** Characteristics of grandparents of children recently diagnosed with cancer (grandparents of patients) and comparison group (grandparents of survivors).

	Grandparents	Comparison
*N*	41	133
Grandparents related characteristics		
Age		
Mean (SD)	67.7 (6.3)	72.3 (7.3)
Range	55–80	56–95
Gender		
Female (%)	24 (58.5)	79 (59.9)
Male (%)	17 (41.5)	53 (40.1)
Nationality		
Swiss (%)	33 (86.8)	110 (84.0)
Non‐Swiss (%)	5 (13.2)	21 (16.0)
Educational level^a^		
Low (%)	1 (2.7)	24 (18.3)
Middle (%)	21 (56.8)	68 (51.9)
High (%)	15 (40.5)	39 (29.8)
Household income level^b^		
≤ 6000 CHF (%)	13 (44.8)	52 (46.9)
> 6000 CHF (%)	16 (55.2)	59 (53.1)
Employment status		
Employed (%)	15 (39.5)	16 (12.3)
Unemployed/retired (%)	23 (60.5)	114 (87.7)
Relationship status		
Partnership (%)	34 (89.5)	110 (85.9)
Single (%)	4 (10.5)	18 (14.1)
Child related characteristics		
Age at diagnosis^c^		
Mean (SD)	6.1 (4.6)	4.8 (3.9)
Range	0–15	0–16
Gender of the child^c^		
Female (%)	12 (63.1)	24 (38.1)
Male (%)	7 (36.8)	39 (61.9)
Total number of grandchildren		
≤ 2 (%)	16 (43.2)	24 (18.2)
> 2 (%)	21 (56.8)	108 (81.8)
Kinship		
Maternal grandmother (%)	13 (31.7)	53 (40.1)
Maternal grandfather (%)	13 (31.7)	35 (26.5)
Paternal grandmother (%)	11 (26.8)	26 (19.7)
Paternal grandfather (%)	4 (9.8)	18 (13.6)
Hospital proximity		
< 0.5 h (%)	10 (27.8)	n.a.
0.5–1 h (%)	19 (52.8)	
1–1.5 h (%)	2 (5.6)	
More than 1.5 h (%)	5 (13.9)	
Cancer related characteristics^c^		
Time since diagnosis		
Mean (SD)	3 months (0.8)	6.7 years (2.3)
Type of cancer		
Leukemia/lymphoma (%)	10 (52.6)	32 (50.8)
Other types of cancer (%)	9 (48.7)	31 (49.2)
Cancer treatment		
Chemotherapy (%)	6 (31.6)	30 (47.6)
Combination/surgery/radiotherapy (%)	13 (68.4)	33 (52.4)

Abbreviations: n.a., not applicable; SD, standard deviation.

^a^ Low (compulsory school) vs. middle (vocational training or high school graduation) vs. high (secondary or university education).

^b^ Net income of the household in Swiss Francs (CHF).

^c^ Missing data: 1 child in the grandparents group; 3 children in the comparison group. Counts are per child, not per grandparent, as a child may have multiple enrolled grandparents.

For the comparison group (grandparents of survivors), 1188 families were contacted. Of these, 221 families provided the contact information, and 133 grandparents (59.9% females) of 66 families/survivors ultimately completed the questionnaire (Table 1). They were largely similar to the grandparents of patients but slightly older (mean 72.3 years, SD 7.3), and thus also more were retired or not employed (87.7%). Survivors were predominantly males (61.9%), on average 4.8 years old at diagnosis, and had a mean time since diagnosis of 6.7 years. Approximately half of survivors had a diagnosis of leukemia or lymphoma (50.8%). Of the patients, 47.6% had received chemotherapy alone, whereas 52.4% had received a combination of surgery, radiotherapy, or a combination of therapies.

### 3.1 Longitudinal Changes of Psychological Distress in Grandparents (Aim 1)

Mean distress scores of grandparents were below norm values at all time points (GSI 45.6–48.0, depression 47.5–49.0, somatization 47.1–49.0, anxiety 45.2–46.8) (Table 2). Mean GSI score increased from 45.6 at T1 to 46.9 at T2 (p 0.098). Otherwise, mean depression, anxiety, and somatization scores did not change over the 2 years (Table S1 for crude and gender‐adjusted models).

**TABLE 2:** Longitudinal changes of psychological distress.

	Mean T1	Mean T2	T1–T2 *p*	Mean T3	T1–T3 *p*	Mean T4	T1–T4 *p*	*p*‐trend^a^
Global severity index	45.6	46.9	0.098	46.6	0.519	48.0	0.520	0.419
Depression	47.5	48.1	0.319	48.1	0.535	49.0	0.608	0.795
Somatization	47.1	48.5	0.180	48.0	0.711	49.0	0.497	0.567
Anxiety	45.9	45.2	0.727	45.7	0.746	46.8	0.544	0.944

Abbreviations: T1, at 3 months; T2, at 6 months; T3, at 12 months; T4, at 24 months.

^a^ Linear mixed models using GSI, depression, somatization and anxiety as outcome, timepoints as predictor, and individual as cluster variable.

**FIGURE 1:**
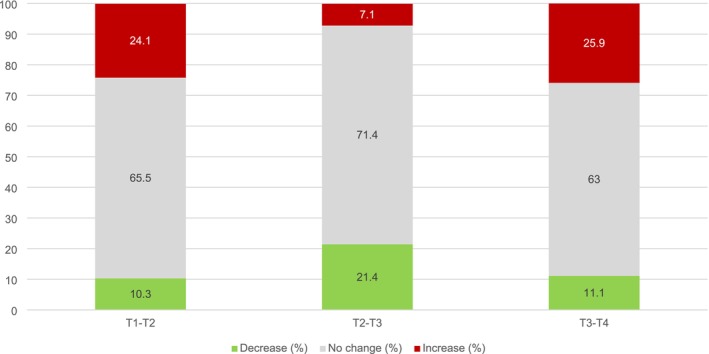
Proportion of grandparents exhibiting significant global symptom index (GSI) changes based on the Reliable Change Index (RCI) over time. T1 at 3 months; T2 at 6 months; T3 at 12 months; T4 at 24 months.

Between T1 and T2, 24.1% showed a reliable increase in distress, 10.3% showed a reliable decrease, and 65.5% reported no change (Figure 1). Between T2 and T3, the proportion of participants with increased distress dropped to 7.1%, whereas 21.4% experienced a decrease and 71.4% reported no change. From T3 to T4, the proportion of participants with increased distress rose again to 25.9%, with 11.1% showing a decrease and 63.0% reporting no change.

At each timepoint, 17.6% (*N* = 6) to 21.6% (*N* = 8) of grandparents met the criteria for clinically significant distress (T1: 21.6%, T2: 18.7%, T3: 17.6%, T4: 19.4%) (Figure S2).

### 3.2 Determinants of Psychological Distress (Aim 2)

Sociodemographic and child‐related characteristics associated with GSI or a specific symptom domain are presented in Table 3 and Tables S2–S4. Grandparents who lived between 0.5 and 1 h (*β*: −5.89; 95% CI −11.98 to 0.20; *p*: 0.058) or between 1 and 1.5 h (*β*: −13.27; 95% CI −26.15 to −1.10; *p*: 0.033) from the child's hospital showed lower GSI scores than grandparents who lived < 0.5 h (Table 3). Grandparents who lived more than 0.5 h from the child's hospital also showed lower depression scores (0.5–1 h: *β*: −5.19; 95% CI −10.55 to 0.17; *p*: 0.058; 1–1.5 h: *β*: −9.97; 95% CI −21.17 to 1.23; *p*: 0.081: more than 1.5 h: *β*: −6.55; 95% CI −14.26 to 1.17; *p*: 0.096 (Table S2)). Grandfathers showed higher somatization scores than grandmothers (*β*: 4.00; 95% CI −0.33 to 0.34; *p*: 0.071) (Table S3). However, the kinship variable showed that these were only for the maternal grandfathers (*β*: 6.47; 95% CI −1.09 to 14.03; *p*: 0.094). Older grandparents showed lower anxiety scores (*β*: −0.31; 95% CI −0.61 to 0.00; *p*: 0.051) and also those who lived 0.5–1 h (*β*: −5.50, 95% CI −10.03 to −0.97; *p*: 0.017) or 1–1.5 h (*β*: −9.86; 95% CI −19.37 to −0.35; *p*: 0.042) from the child's hospital (Table S4).

**TABLE 3:** Determinants of psychological distress (using global severity index).

	Mean^a^	95% Confidence interval^a^	Beta‐coefficient	95% Confidence interval	*p*
Grandparent related characteristics					
Age	—^b^	—^b^	−0.24	−0.68, 0.20	0.288
Gender					
Female—ref.	45.18	41.77, 48.59			
Male	47.48	43.33, 51.62	2.30	−3.07, 7.66	0.401
Nationality					
Swiss—ref.	46.70	43.80, 49.62			
Non‐Swiss	44.01	36.49, 51.52	−2.70	−10.76, 5.36	0.511
Educational level^c^					
Low/middle—ref.	45.13	41.50, 48.76			
High	48.00	43.71, 52.30	2.87	−2.75, 8.50	0.317
Household income level^d^					
≤ 6000 CHF—ref.	45.74	41.39, 50.08			
> 6000 CHF	48.74	44.82, 52.66	3.00	−2.85, 8.86	0.315
Employment status					
Unemployed/retired—ref.	44.86	41.45, 48.27			
Employed	48.65	44.43, 52.86	−3.79	−9.21, 1.64	0.171
Relationship status					
Partnership—ref.	46.73	43.88, 49.59			
Single	43.08	34.69, 51.48	1.83	−1.44, 2.90	0.420
Child‐related characteristics					
Age	—^b^	—^b^	0.09	−0.51, 0.690	0.770
Gender					
Female—ref.	46.86	43.24, 50.47			
Male	45.28	40.69, 49.86	−1.58	−7.42, 4.26	0.596
Total number of grandchildren					
≤ 2—ref.	47.64	43.59, 51.69			
> 2	44.77	41.19, 48.35	−2.87	−8.27, 2.53	0.298
Kinship					
Maternal grandmother—ref.	45.12	40.43, 49.81			
Maternal grandfather	48.87	44.03, 53.71	3.75	−2.99, 0.49	0.276
Paternal grandmother	45.25	40.19, 50.31	0.13	−6.77, 7.03	0.970
Paternal grandfather	43.38	35.07, 51.69	−1.74	−11.28, 7.81	0.721
Hospital proximity					
< 0.5 h (%—ref.)	51.02	46.11, 55.94			
0.5–1 h	45.14	41.54, 48.74	−5.89	−11.98, 0.20	0.058*
1–1.5 h	37.30	25.88, 48.92	−13.27	−26.15, −1.10	0.033**
More than 1.5 h	45.91	38.75, 53.08	−5.11	−13.80, 3.58	0.249
Cancer related characteristics					
Type of cancer					
Leukemia/lymphoma—ref.	46.43	42.71, 50.16			
Other types of cancer	46.00	41.69, 50.40	2.94	−6.21, 5.33	0.882
Cancer treatment					
Chemotherapy—ref.	46.65	42.24, 51.06			
Combination/surgery/radiotherapy	45.97	42.24, 49.69	−0.69	−6.45, 5.08	0.816

^a^ Marginal mean and confidence interval.

^b^ Not computed because there are no categories (continuous variable).

^c^ Low (compulsory school) vs. middle (vocational training or high school graduation) vs. high (secondary or university education).

^d^ Net income of the household in CHF.

*p ≤ 0.10; **p ≤ 0.05.

### 3.3 Comparison of Psychological Distress of Grandparents and the Comparison Group (Aim 3)

Grandparents showed higher depression scores at T2 and T4 than the comparison group in both crude and age‐adjusted models (Table 4). The other measurement time points or domains did not differ. The proportion of clinically significant distress was also higher among grandparents (18%–22%) than the comparison group (9%).

**TABLE 4:** Comparison of psychological distress in grandparents of children recently diagnosed with cancer and comparison group.

	Mean of comparison	T1 vs. comparison			T2 vs. comparison			T3 vs. comparison			T4 vs. comparison		
		Mean at T1	Crude *p*	Age adjusted *p*	Mean at T2	Crude *p*	Age adjusted *p*	Mean at T3	Crude *p*	Age adjusted *p*	Mean at T4	Crude *p*	Age adjusted *p*
Global severity index	45.7	45.6	0.804	0.855	46.9	0.463	0.188	46.6	0.955	0.330	48.0	0.487	0.125
Depression	45.9	47.5	0.134	0.064	48.1	0.035	0.015	48.1	0.106	0.013	49.0	0.028	0.006
Somatization	48.5	47.1	0.153	0.910	48.5	0.247	0.723	48.0	0.140	0.945	49.0	0.268	0.516
Anxiety	45.5	45.9	0.148	0.310	45.2	0.112	0.874	45.7	0.244	0.746	46.8	0.160	0.473

Abbreviations: T1, at 3 months; T2, at 6 months; T3, at 12 months; T4, at 24 months.

## 4 Discussion

Our study examined psychological distress among grandparents following their grandchild's cancer diagnosis. Grandparents of children with a recent diagnosis reported at 3–6 months and at 12–24 months after diagnosis slightly higher levels of depression compared to comparisons (grandparents of survivors). However, overall distress levels remained below population norms, suggesting that most grandparents are coping well. Longer travel time to the hospital (hospital proximity), older age, and kinship were associated with psychological distress.

### 4.1 Longitudinal Changes of Psychological Distress

Our findings showed that grandparents' psychological distress increased around 6 months after diagnosis, coinciding with the peak period of familial stress [21]. Previous studies showed that parental emotional stress increased during the first 6 months after diagnosis [21] and that parents reported higher stress levels when compared to parents of healthy children [22]. Our data extend these findings to grandparents. These observations suggest that grandparents also experience psychological burden not only in the immediate aftermath of diagnosis but also after the end of therapy. Grandparents may experience psychological distress with some delay compared to parents, partly due to the dynamics of their caregiving role. Following a cancer diagnosis, grandparents often assume a supportive role for the entire family [2]. Parents who focus intensely on caring for the child, may initially be unaware of their own emotional strain and begin to process their experiences only after the acute phase subsides [23]. This delay in parental processing may also affect grandparents who often suppress their own emotions while supporting others. The triple concern for their grandchild, their own child (parent of the patient) and the siblings has been associated with delayed recognition of distress among grandparents [7, 24]. Another possible explanation for this delayed emotional response is that grandparents may initially show strength to support the parents [25], but process their own emotions only later in the childhood cancer trajectory [26]. This often happens when parents have gained more emotional and practical support and are experiencing less stress.

The increase in psychological distress observed at 12–24 months may be partially explained by caregiver fatigue following the intensive treatment phase. Caregiver fatigue refers to a state of physical and emotional exhaustion resulting from sustained caregiving demands, frequent medical procedures, and prolonged exposure to illness‐related stressors [27]. Depending on the cancer type and severity, the initial months following diagnosis often involve intensive treatment protocols, which can lead to significant caregiver burden and cumulative fatigue, particularly towards the end of therapy [28]. Caregiver fatigue has been shown to contribute to higher psychological distress [27]. Although no studies have specifically investigated caregiver fatigue among grandparents, it is reasonable to assume that similar processes may occur among those who are actively involved in the care of the child. In the absence of grandparent‐specific data, existing evidence from parents provides the most relevant basis for extrapolation. Furthermore, older grandparents may experience additional stress due to pre‐existing chronic health conditions, and the concurrent management of their own health needs while providing care may further increase the risk of psychological distress [29].

### 4.2 Determinants of Psychological Distress

We identified some sociodemographic characteristics of grandparents associated with the severity of psychological distress, namely, hospital proximity, older age, and kinship. First, longer travel time to the hospital may limit grandparents' ability to provide instrumental support, such as caring for siblings or helping with household tasks, reducing their overall involvement [30]. Studies have shown that grandparents who take on more childcare responsibilities tend to report more severe symptoms of depression and anxiety [31]. The stress of caregiving can lead to heightened anxiety, especially among grandparents who view their caregiving responsibilities as a burden. Second, older age was associated with lower anxiety, which aligns with previous research showing that older grandparents report less health‐related anxiety and fewer emotional concerns for themselves and their grandchildren [32]. Higher resilience in older adults may further explain the lower levels of anxiety [33]. They also tend to have less caregiving involvement, which has been linked to lower stress and better coping ability [34]. Third, maternal grandfathers were found to have higher somatization scores. Studies have shown that the maternal side is more likely to be involved than the paternal side of the family [35]. Another possible explanation is that older people often struggle with age‐related physical problems, and the stress of a grandchild's serious illness may exacerbate these problems [36]. Due to cultural and generational norms regarding the expression of distress, older people (especially men) tend to show emotional pain through physical symptoms rather than discussing or processing their feelings openly [37].

### 4.3 Comparative Analysis of Psychological Distress

Distress scores were similar between grandparents and comparisons. However, for depression, grandparents reported higher levels than comparisons at 6 and 24 months after the grandchild's cancer diagnosis. In our previous study [14], grandparents of survivors had similar psychological distress levels to the matched Swiss general population, indicating that most grandparents maintained mental health within normative ranges and do not require specialized psychological intervention [8]. We confirmed these findings in our cohort study of grandparents whose grandchildren were recently diagnosed with cancer. Distress levels stayed within normal ranges across different domains (anxiety, depression, and somatization) and at multiple time points, even though many children were still receiving intensive cancer treatment. The longitudinal design of this study, with repeated measurements at several time points, ensured a robust assessment of psychological distress, reinforcing the findings that this population does not exhibit clinically relevant mental health problems. Nevertheless, our findings warrant a more nuanced interpretation. On the one hand, these lower values may reflect those grandparents who can cope well with the difficult situation they experience. Previous studies have shown that, compared to younger individuals, older people exhibit lower overall psychological distress [16], enhanced emotional stability and problem‐solving capabilities during stressful situations [34]. On the other hand, the increases observed 3–6 months and again at 12–24 months, highlighted critical periods of vulnerability. As older adults may typically have low baseline distress levels, acute increases in symptoms are indicative of psychological strain and a potential risk for developing mental health disorders [34].

### 4.4 Strengths and Limitations

To our knowledge, our study is the first in Europe, and one of the few internationally to focus on grandparents of children with cancer. It is also, to date, the only cohort analysis conducted in this population. Previous studies were cross‐sectional surveys [4, 11, 38], which could not capture the dynamic nature of psychosocial outcomes during a critical event. Our findings provided novel findings into the temporal development of psychological distress in this population. Finally, our study was a multicenter study from eight pediatric oncology centers in the country, across different language regions (French‐, German‐, and Italian‐speaking areas), and thereby maximizing enrollment in a relatively rare disease.

However, there are critical limitations that need to be considered in the interpretation of our findings. First, despite multicenter enrollment, the overall sample size was small, and several grandparents did not complete the questionnaires at all measurement points. This limits the statistical power of our findings and the feasibility of conducting complex statistical analyses. To account for this, we used linear mixed models, which are supported in the literature as efficient for handling missing data and potentially appropriate for small samples [39]. The limited participation may reflect selection bias, such that grandparents experiencing elevated stress levels or significant burden may have been less likely to enroll or more likely to withdraw prematurely. This pattern could result in an underestimation of distress levels among grandparents, as our sample may primarily include those with greater personal resources or lower baseline stress. The low response rate might also have been influenced by the COVID‐19 pandemic. For an extended period, only parents were allowed in pediatric wards, restricting direct access to grandparents. Consequently, study materials were provided to parents to relay to grandparents, which may have contributed to reduced participation. To mitigate this, we extended the enrollment period of the cohort. Second, grandparents were recruited indirectly through parents of childhood cancer patients, introducing potential selection bias. It is likely that those who participated were grandparents with stronger relationships with the parents or children, potentially leading to an overestimation of psychological distress. Third, although we observed changes over time, our models did not include time‐varying covariates such as social support or financial status, which may influence distress levels over the 2‐year period. An addition of another cluster to account for family units or grandparent couples would have been preferable, given that many psychosocial variables are likely correlated within families. However, the available sample size was insufficient to include time‐varying covariates or to implement a three‐level hierarchical model, which is another limitation of the current analyses. Given all the limitations, our findings should be considered exploratory.

### 4.5 Clinical Implications and Future Research

Although the levels of psychological distress in grandparents are within population norms, those with clinically significant distress may still require psychological support. In the current standard of psychosocial care for childhood cancer, the focus is mainly on the patient, parents, and siblings, with limited consideration given to grandparents [40, 41]. Few programs exclusively for grandparents can be found in the literature [42]. However, none yet exist in Europe and specifically in Switzerland. Previous studies showed that access to support services that are tailored to the specific experiences of grandparents can help to reduce mental distress and build resilience [4, 25, 43]. Our study emphasizes the importance of providing appropriate and tailored information and support to grandparents when needed.

Future research should focus on identifying the information and support needs of grandparents following their grandchild's diagnosis of cancer. Grandparents are known to be a resource for parents going through stressful times. Studies should be conducted to identify their role and the support that grandparents provide for the family. More studies with larger sample size are needed to investigate more complex associations to understand the psychosocial impact of a childhood cancer diagnosis.

## 5 Conclusion

In this exploratory study, most grandparents exhibited psychological distress within normal ranges, suggesting generally adaptive coping following their grandchild's cancer diagnosis. However, distress levels increased at 6 months and 1 year post‐diagnosis. Compared to grandparents of childhood cancer survivors, these grandparents reported higher depression levels within 2 years after diagnosis. Factors such as distance to the child's treatment hospital, age, and kinship influenced psychological distress. Psychological support should be made available to those at risk of heightened distress.

## Author Contributions

**Barbara Gantner:** conceptualization, methodology, data collection, analysis, writing – original draft, writing – review and editing. **Cristina Priboi:** conceptualization, methodology, data collection, writing – review and editing, project administration. **Anica Ilic:** conceptualization, methodology, data collection, writing – review and editing, project administration. **Freimut H. Schilling:** data collection, writing – review and editing. **Ahmed Farrag:** data collection, writing – review and editing. **Katrin Scheinemann:** conceptualization, data collection, writing – review and editing, funding acquisition. **Marc Ansari:** data collection, writing – review and editing. **Jeanette Greiner:** data collection, writing – review and editing. **Nicolas von der Weid:** data collection, writing – review and editing. **Pierlugi Brazzola:** data collection, writing – review and editing. **Manuel Diezi:** data collection, writing – review and editing. **Elena Lemmel:** data collection, writing – review and editing. **Pauline Holmer:** conceptualization, methodology, data collection, writing – review and editing, project administration. **Peter Francis Raguindin:** conceptualization, methodology, data collection, analysis, writing – review and editing, project administration, supervision. **Gisela Michel:** conceptualization, data collection, writing – review and editing, project administration, funding acquisition, supervision.

## Funding

The work was supported by the Swiss National Science Foundation (grant number 10001C_182129/1).

## Ethics Statement

Approved by the Ethics Committee of Northwest and Central Switzerland (EKNZ 2020‐01409, 26 August 2020). The research was conducted in accordance with the principles of the Declaration of Helsinki. The study is compliant with the Swiss Human Research Act (810.30 Federal Act of 30 September 2011 on Research involving Human Beings) and Federal Regulations on Data Protection (235.1 Federal Act on Data Protection of 25 September 2020).

## Consent

All participants provided informed consent for the anonymized publication of the data collected during the study. No identifying information has been disclosed.

## Conflicts of Interest

The authors declare no conflicts of interest.

## Supporting information


**Figure S1:** cam471774‐sup‐0001‐Supinfo.docx.
**Figure S2:** cam471774‐sup‐0001‐Supinfo.docx.
**Table S1:** cam471774‐sup‐0001‐Supinfo.docx.
**Table S2:** cam471774‐sup‐0001‐Supinfo.docx.
**Table S3:** cam471774‐sup‐0001‐Supinfo.docx.
**Table S4:** cam471774‐sup‐0001‐Supinfo.docx.

## Data Availability

The data that support the findings of this study are available on request from the corresponding author. The data are not publicly available due to privacy or ethical restrictions.
