# Supplementary material for: Longitudinal Analysis of Psychological Distress in Grandparents of Children With Cancer: Results From a Multicenter Cohort Study in Switzerland (GROKids Project)
Source: Cancer Med. 2026 Apr 7;15(4):e71774. doi: 10.1002/cam4.71774 (PMC13056702; doi:10.1002/cam4.71774)
Supplement: Supplementary file 1 — Figure S1: cam471774‐sup‐0001‐Supinfo.docx. Figure S2: cam471774‐sup‐0001‐Supinfo.docx. Table S1: cam471774‐sup‐0001‐Supinfo.docx. Table S2: cam471774‐sup‐0001‐Supinfo.docx. Table S3: cam471774‐sup‐0001‐Supinfo.docx. Table S4: cam471774‐sup‐0001‐Supinfo.docx. [file CAM4-15-e71774-s001.docx]

# Supporting Information

Longitudinal analysis of psychological distress in grandparents of children with cancer: Results from a multicenter cohort study in Switzerland (GROKids Project)

Running head

Longitudinal Psychological Distress in Grandparents

Authors

Barbara GANTNER^1,2^, Cristina PRIBOI^1,3,4,5^, Anica ILIC^1,6^, Freimut H. SCHILLING^7^, Ahmed FARRAG^7,8^, Katrin SCHEINEMANN^1,9^, Marc ANSARI^10,11^, Jeanette GREINER^12^, Nicolas VON DER WEID^13^, Pierlugi BRAZZOLA^14^, Manuel DIEZI^15^, Elena LEMMEL^15^, Pauline HOLMER^1,16^, Peter Francis RAGUINDIN^1^* and Gisela MICHEL^1^*

^1^ Faculty of Health Sciences and Medicine, University of Lucerne, Lucerne, Switzerland

^2^ Consultation-Liaison Psychiatric Service of Lucerne Psychiatric Service in the Children`s Hospital of Central Switzerland, Lucerne, Switzerland

^3^ Veterinary Public Health Institute, University of Bern, Bern, Switzerland

^4^ Institute for Biomedical Ethics, University of Basel, Basel, Switzerland

^5^Faculty of Psychology and Educational Sciences, University of Geneva, Geneva, Switzerland

^6^Institute of Basic Medical Sciences, Faculty of Medicine, University of Oslo, Oslo, Norway

^7^ Division of Pediatric Hematology and Oncology, Department of Pediatrics,

Children’s Hospital of Central Switzerland, Lucerne, Switzerland

^8^ Pediatric Oncology Department, South Egypt Cancer Institute, Assiut University, Assiut, Egypt

^9^ Division of Hematology/Oncology, Children’s Hospital of Eastern Switzerland, St. Gallen, Switzerland

^10^ CANSEARCH Research Platform for Pediatric Oncology and Hematology, Faculty of Medicine, Department of Pediatrics, Gynecology and Obstetrics, University of Geneva, Geneva, Switzerland

^11^ Division of Pediatric Oncology and Hematology, Department of Women, Child and Adolescent, University Geneva Hospitals, Geneva, Switzerland

^12^ Pediatric Oncology-Hematology, Children's Hospital, Cantonal Hospital Aarau, Aarau, Switzerland

^13^ Department of Pediatric Oncology and Hematology, University Children’s Hospital Basel, Basel, Switzerland

^14^ Emato-Oncologia pediatrica, Istituto Pediatrico della Svizzera Italiana, Ospedale Regionale di Bellinzona e Valli, Bellinzona, Switzerland

^15^ Pediatric Hematology and Oncology Unit, Department of Pediatrics, University Hospital Center Lausanne, Lausanne, Switzerland

^16^ Faculty of Human Sciences, Department of Inclusive Education, University of Potsdam, Potsdam, Germany

* Shared last author

Corresponding author

Prof. Dr. Gisela Michel

Faculty of Health Sciences and Medicine

University of Lucerne

Alpenquai 4, 6005 Lucerne

Email: [gisela.michel@unilu.ch](mailto:gisela.michel@unilu.ch)


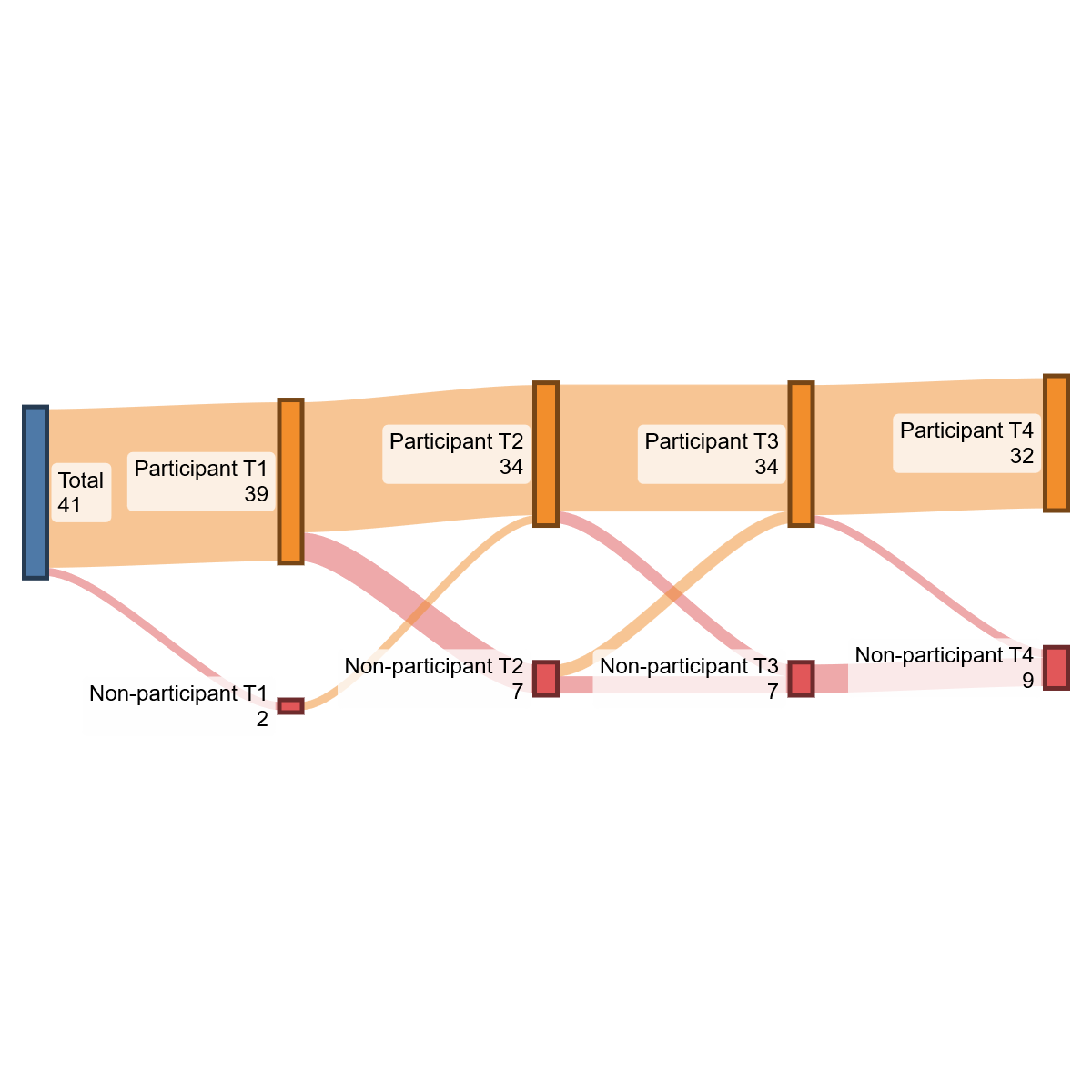


**Figure S1.** Flowchart of study participants

**
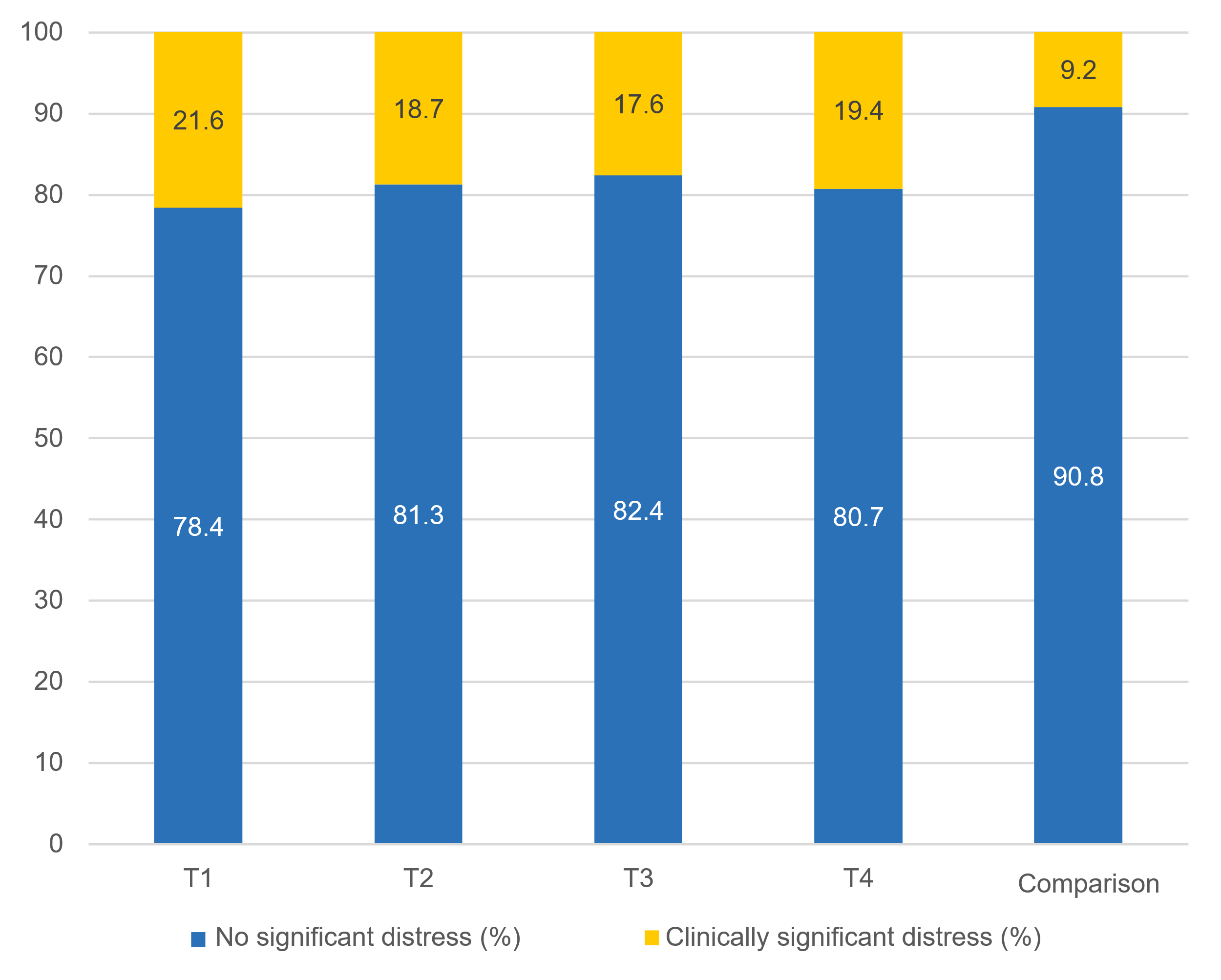
**

**Figure S2**. Proportion of no clinically significant distress among grandparents across time*

*Abbreviations: T1: at 3 months (N=39); T:2 at 6 months (N=34); T3: at 12 months (N=34); T4: at 24 months (N=32); Comparison: grandparents of survivors (N=133).* *Global severity index T score >57 used as cut-off

**Table S1.** Changes in psychological distress (GSI) over time^1^

|  |  |  |  | Crude model | | |  | Gender-adjusted model^3^ | | |
| --- | --- | --- | --- | --- | --- | --- | --- | --- | --- | --- |
|  | Mean Score^2^ | Confidence interval^2^ |  | Beta-coefficient | 95% Confidence interval | p-value |  | Beta-coefficient | 95% Confidence interval | p-value |
| **GSI** |  |  |  |  |  |  |  |  |  |  |
| 3 months (T1) | 45.35 | 42.49 48.20 |  | ref | -- | -- |  | -- | -- | -- |
| 6 months (T2) | 47.13 | 44.18 50.09 |  | 1.78 | -0.31 3.88 | 0.095* |  | 1.77 | -0.33 3.86 | 0.098* |
| 12 months (T3) | 46.04 | 43.13 48.96 |  | 0.70 | -1.35 2.74 | 0.504 |  | 0.67 | -1.37 2.72 | 0.519 |
| 24 months (T4) | 46.06 | 43.10 49.02 |  | 0.71 | -1.39 2.82 | 0.507 |  | 0.69 | -1.42 2.80 | 0.518 |
| **Depression** |  |  |  |  |  |  |  |  |  |  |
| 3 months (T1) | 47.17 | 44.58 49.75 |  | ref | -- | -- |  | -- | -- | -- |
| 6 months (T2) | 48.31 | 45.59 51.04 |  | 1.15 | -1.10 3.39 | 0.318 |  | 1.14 | -1.11 3.39 | 0.319 |
| 12 months (T3) | 47.87 | 45.20 50.54 |  | 0.70 | -1.49 2.89 | 0.532 |  | 0.69 | -1.50 2.89 | 0.535 |
| 24 months (T4) | 47.77 | 45.04 50.49 |  | 0.60 | -1.66 2.86 | 0.604 |  | 0.59 | -1.67 2.86 | 0.608 |
| **Somatization** |  |  |  |  |  |  |  |  |  |  |
| 3 months (T1) | 47.33 | 44.86 49.79 |  | ref | -- | -- |  | -- | -- | -- |
| 6 months (T2) | 48.82 | 46.24 51.40 |  | 1.49 | -0.63 3.61 | 0.167 |  | 1.45 | -0.67 3.56 | 0.180 |
| 12 months (T3) | 47.77 | 45.24 50.31 |  | 0.45 | -1.62 2.52 | 0.670 |  | 0.39 | -1.68 2.46 | 0.711 |
| 24 months (T4) | 48.11 | 45.53 50.69 |  | 0.78 | -1.35 2.92 | 0.471 |  | 0.74 | -1.39 2.87 | 0.497 |
| **Anxiety** |  |  |  |  |  |  |  |  |  |  |
| 3 months (T1) | 45.60 | 43.36 47.84 |  | ref | -- | -- |  | -- | -- | -- |
| 6 months (T2) | 45.23 | 42.87 47.60 |  | -0.37 | -2.360 1.62 | 0.717 |  | -0.36 | -2.35 1.69 | 0.727 |
| 12 months (T3) | 45.26 | 42.94 47.57 |  | -0.34 | -2.29 1.60 | 0.729 |  | -0.32 | -2.27 1.62 | 0.746 |
| 24 months (T4) | 44.98 | 42.61 47.34 |  | -0.62 | -2.63 1.38 | 0.534 |  | -0.61 | -2.61 1.40 | 0.544 |

*p ≤ 0.10, ** p ≤ 0.05

^1^Linear mixed models using GSI, depression, Somatization and Anxiety as outcome, timepoints as predictor, and individual as cluster variable. Beta-coefficient signifies the change over time with T1 (3 months) as reference

^2^Marginal means and confidence interval

^3^ Linear mixed models adjusted for gender as a covariate in fixed effects.

Abbreviations: GSI, global severity index; ref, reference group

**Table S2.** Determinants of depression

|  | Mean^1^ | Confidence  Interval^1^ | Beta coefficient | 95% Confidence  interval | p-value |
| --- | --- | --- | --- | --- | --- |
| **Grandparents related characteristics** |  |  |  |  |  |
| Age | ---^2^ | ---^2^ | -0.32 | -0.70 0.07 | 0.104 |
| Gender |  |  |  |  |  |
| - Female - ref | 47.59 | 44.58 50.96 |  |  |  |
| - Male | 47.98 | 44.34 51.62 | 0.39 | -4.33 5.11 | 0.871 |
| Nationality |  |  |  |  |  |
| - Swiss - ref | 48.75 | 46.27 51.24 |  |  |  |
| - Non-Swiss | 43.73 | 37.28 50.18 | -5.02 | -11.93 1.90 | 0.154 |
| Educational level^3^ |  |  |  |  |  |
| - Low/Middle - ref | 47.69 | 44.52 50.86 |  |  |  |
| - High | 49.15 | 45.44 52.86 | 1.47 | -3.42 6.35 | 0.556 |
| Household income level^4^ |  |  |  |  |  |
| - <= 6000 CHF - ref | 46.49 | 42.45 50.54 |  |  |  |
| - > 6000 CHF | 50.06 | 46.41 53.71 | 3.57 | -1.88 9.01 | 0.199 |
| Employment status |  |  |  |  |  |
| - Unemployed/retired - ref | 46.60 | 43.64 49.55 |  |  |  |
| - Employed | 50.41 | 46.76 54.05 | -3.81 | -8.50 0.88 | 0.112 |
| Relationship status |  |  |  |  |  |
| - Partnership-ref | 48.39 | 45.88 50.89 |  |  |  |
| - Single | 45.61 | 38.23 52.99 | 1.39 | -2.51 5.29 | 0.485 |

**Table S2, continued**

|  | Mean^1^ | Confidence  Interval^1^ | Beta coefficient | 95% Confidence  interval | p-value |
| --- | --- | --- | --- | --- | --- |
| **Child related characteristics** |  |  |  |  |  |
| Age | ---^2^ | ---^2^ | -0.04 | -0.57 0.49 | 0.882 |
| Gender |  |  |  |  |  |
| - Female-ref | 47.22 | 44.05 50.39 |  |  |  |
| - Male | 48.15 | 44.01 52.19 | 0.93 | -4.21 6.06 | 0.724 |
| Total number of grandchildren |  |  |  |  |  |
| - <=2- ref | 48.60 | 45.13 52.08 |  |  |  |
| - > 2 | 47.00 | 43.92 50.09 | -1.60 | -6.25 3.05 | 0.500 |
| Kinship |  |  |  |  |  |
| - Maternal grandmother - ref | 47.72 | 43.57 51.88 |  |  |  |
| - Maternal grandfather | 49.07 | 44.81 53.35 | 1.36 | -4.60 7.32 | 0.656 |
| - Paternal grandmother | 47.43 | 42.97 51.90 | -0.29 | -6.39 5.81 | 0.926 |
| - Paternal grandfather | 44.78 | 37.51 52.06 | -2.94 | -11.32 5.44 | 0.492 |
| Hospital proximity |  |  |  |  |  |
| - < 0.5 hour - ref | 52.22 | 47.91 56.54 |  |  |  |
| - 0.5 hour to 1 hour | 47.03 | 43.86 50.21 | -5.19 | -10.55 0.17 | 0.058* |
| - 1 hour to 1.5 hours | 42.25 | 31.92 52.59 | -9.97 | -21.17 1.23 | 0.081* |
| - > than 1.5 hours | 45.68 | 39.29 52.07 | -6.55 | -14.26 1.17 | 0.096* |
| **Cancer related characteristics** |  |  |  |  |  |
| Type of cancer |  |  |  |  |  |
| - Leukemia/ Lymphoma -ref | 44.82 | 44.55 51.09 |  |  |  |
| - Other types of cancer | 47.23 | 43.36 51.10 | -0.59 | -5.66 4.48 | 0.820 |
| Cancer treatment |  |  |  |  |  |
| - Chemotherapy - ref | 47.79 | 43.92 51.66 |  |  |  |
| - Combination/ surgery / radiotherapy | 47.48 | 44.15 50.69 | -3.37 | -5.44 4.70 | 0.887 |

*p ≤ 0.10, ** p ≤ 0.05

^1^ *marginal mean and confidence interval*

^2^ *not computed because there were no categories*

^3^ *low (compulsory school) vs. middle (vocational training or high school graduation) vs. high (secondary or university education)*

^4^*Net income of the household in Swiss Francs (CHF)*

**Table S3.** Determinants of somatization

|  | Mean^1^ | Confidence Interval^1^ | Beta coefficient | 95% Confidence interval | p-value |
| --- | --- | --- | --- | --- | --- |
| **Grandparent related characteristics** |  |  |  |  |  |
| Age | ---^2^ | ---^2^ | -0.02 | -0.38 0.34 | 0.909 |
| Gender |  |  |  |  |  |
| - Female - ref | 46.35 | 43.59 49.11 |  |  |  |
| - Male | 50.35 | 47.01 53.69 | 4.00 | -0.33 8.33 | 0.071* |
| Nationality |  |  |  |  |  |
| - Swiss - ref | 47.77 | 45.37 50.16 |  |  |  |
| - Non-Swiss | 48.85 | 42.65 55.05 | 1.08 | -5.56 7.73 | 0.749 |
| Educational level^3^ |  |  |  |  |  |
| - Low/Middle - ref | 47.20 | 44.25 50.14 |  |  |  |
| - High | 48.23 | 44.77 51.68 | 1.03 | -3.52 5.57 | 0.657 |
| Household income level^4^ |  |  |  |  |  |
| - <= 6000 CHF - ref | 48.36 | 44.58 52.13 |  |  |  |
| - > 6000 CHF | 48.94 | 45.54 52.34 | 0.58 | -4.50 5.66 | 0.822 |
| Employment status |  |  |  |  |  |
| - Unemployed/retired - ref | 47.11 | 44.26 49.96 |  |  |  |
| - Employed | 49.13 | 45.61 52.64 | -2.02 | -6.55 2.51 | 0.382 |
| Relationship status |  |  |  |  |  |
| - Partnership-ref | 48.16 | 45.81 50.52 |  |  |  |
| - Single | 45.67 | 38.74 52.61 | 1.25 | -2.42 4.91 | 0.505 |

**Table S3, continued**

|  | | Mean^1^ | Confidence Interval^1^ | Beta coefficient | 95% Confidence interval | p-value |  |
| --- | --- | --- | --- | --- | --- | --- | --- |
| **Child related characteristics** |  |  | | | | | |
| Age | | ---^2^ | ---^2^ | 0.21 | -0.30 0.71 | 0.423 |  |
| Gender | |  |  |  |  |  |  |
| - Female-ref | | 49.68 | 46.75 52.60 |  |  |  |  |
| - Male | | 46.78 | 42.99 50.48 | -2.94 | -7.96 1.81 | 0.225 |  |
| Total number of grandchildren | |  |  |  |  |  |  |
| - <=2 - ref | | 47.73 | 44.34 51.12 |  |  |  |  |
| - > 2 | | 47.62 | 44.61 50.63 | -0.11 | -4.65 4.43 | 0.962 |  |
| Kinship | |  |  |  |  |  |  |
| - Maternal grandmother - ref | | 45.98 | 42.26 49.70 |  |  |  |  |
| - Maternal grandfather | | 52.00 | 48.18 55.82 | 6.02 | 0.69 11.35 | 0.027** |  |
| - Paternal grandmother | | 46.79 | 42.80 50.78 | 0.81 | -4.65 6.26 | 0.772 |  |
| - Paternal grandfather | | 45.53 | 39.01 52.05 | -0.45 | -4.65 7.06 | 0.906 |  |
| Hospital proximity | |  |  |  |  |  |  |
| - < 0.5 hour - ref | | 49.39 | 45.07 53.72 |  |  |  |  |
| - 0.5 hour to 1 hour | | 47.43 | 44.25 50.61 | -1.96 | -7.33 3.41 | 0.474 |  |
| - 1 hour to 1.5 hours | | 42.25 | 31.96 52.53 | -7.02 | -18.31 4.01 | 0.209 |  |
| - > than 1.5 hours | | 50.12 | 43.75 56.49 | 0.73 | -6.97 8.43 | 0.853 |  |
| **Cancer related characteristics** |  |  | | | | | |
| Type of cancer | |  |  |  |  |  |  |
| - Leukemia/ Lymphoma -ref | | 48.94 | 45.88 52.01 |  |  |  |  |
| - Other types of cancer | | 48.03 | 44.40 51.66 | -0.91 | -5.66 3.84 | 0.706 |  |
| Cancer treatment | |  |  |  |  |  |  |
| - Chemotherapy - ref | | 49.06 | 45.44 52.69 |  |  |  |  |
| - Combination/ surgery / radiotherapy | | 48.21 | 45.14 51.27 | -0.86 | -5.60 3.89 | 0.724 |  |

*p ≤ 0.10, ** p ≤ 0.05

^1^ *marginal mean and confidence interval*

^2^ *not computed because there were no categories*

^3^ *low (compulsory school) vs. middle (vocational training or high school graduation) vs. high (secondary or university education)*

^4^*Net income of the household in Swiss Francs (CHF)*

**Table S4.** Determinants of anxiety

|  | Mean^1^ | Confidence  Interval^1^ | Beta coefficient | 95% Confidence  interval | p-value |
| --- | --- | --- | --- | --- | --- |
| **Grandparent related characteristics** |  |  |  |  |  |
| Age | ---^2^ | ---^2^ | -0.31 | -0.61 0.00 | 0.051* |
| Gender |  |  |  |  |  |
| - Female - ref | 45.94 | 43.37 48.51 |  |  |  |
| - Male | 44.32 | 41.22 47.43 | -1.62 | -5.65 2.41 | 0.431 |
| Nationality |  |  |  |  |  |
| - Swiss - ref | 46.06 | 43.90 48.21 |  |  |  |
| - Non-Swiss | 42.62 | 37.02 48.21 | -3.44 | -9.44 2.56 | 0.261 |
| Educational level^3^ |  |  |  |  |  |
| - Low/Middle - ref | 45.53 | 42.81 48.26 |  |  |  |
| - High | 46.16 | 42.97 49.35 | 0.63 | -3.57 4.83 | 0.769 |
| Household income level^4^ |  |  |  |  |  |
| - <= 6000 CHF - ref | 45.81 | 42.42 49.19 |  |  |  |
| - > 6000 CHF | 46.45 | 43.39 49.51 | 0.64 | -3.92 5.21 | 0.782 |
| Employment status |  |  |  |  |  |
| - Unemployed/retired - ref | 44.39 | 41.85 46.94 |  |  |  |
| - Employed | 47.48 | 44.34 55.62 | -3.09 | -7.13 0.96 | 0.135 |
| Relationship status |  |  |  |  |  |
| - Partnership-ref | 45.87 | 43.73 49.63 |  |  |  |
| - Single | 43.29 | 36.96 49.63 | 1.29 | -2.05 4.63 | 0.449 |

**Table S4, continued**

|  | | Mean^1^ | | Confidence  Interval^1^ | Beta coefficient | 95% Confidence  interval | p-value |
| --- | --- | --- | --- | --- | --- | --- | --- |
| **Child related characteristics** |  | |  | | | | |
| Age | | ---^2^ | | ---^2^ | -0-03 | -0.49 0.63 | 0.891 |
| Gender | |  | |  |  |  |  |
| - Female-ref | | 44.96 | | 42.26 47.66 |  |  |  |
| - Male | | 45.97 | | 42.51 49.43 | 1.01 | -3.38 5.40 | 0.652 |
| Total number of grandchildren | |  | |  |  |  |  |
| - <=2- ref | | 46.66 | | 43.57 49.58 |  |  |  |
| - > 2 | | 44.53 | | 41.79 47.28 | -2.13 | -6.27 2.00 | 0.312 |
| Kinship | |  | |  |  |  |  |
| - Maternal grandmother - ref | | 45.90 | | 42.31 49.48 |  |  |  |
| - Maternal grandfather | | 44.79 | | 41.10 48.47 | -1.11 | -6.25 4.04 | 0.673 |
| - Paternal grandmother | | 45.98 | | 42.12 49.83 | 0.08 | -5.81 5.34 | 0.976 |
| - Paternal grandfather | | 42.95 | | 36.68 49.23 | -2.94 | -10.17 4.29 | 0.425 |
| Hospital Proximity | |  | |  |  |  |  |
| - < 0.5 hour - ref | | 49.76 | | 46.11 53.41 |  |  |  |
| - 0.5 hour to 1 hour | | 44.26 | | 41.57 46.94 | -5.50 | -10.03 -0.97 | 0.017** |
| - 1 hour to 1.5 hours | | 39.90 | | 31.12 48.68 | -9.86 | -19.37 -0.35 | 0.042** |
| - > 1.5 hours | | 44.98 | | 39.56 50.93 | -4.78 | -11.32 1.75 | 0.151 |
| **Cancer related characteristics** |  | |  | | | | |
| Type of cancer | |  | |  |  |  |  |
| - Leukemia/ Lymphoma -ref | | 45.06 | | 42.26 47.86 |  |  |  |
| - Other types of cancer | | 45.73 | | 42.42 49.05 | 0.68 | -3.66 1.57 | 0.760 |
| Cancer treatment | |  | |  |  |  |  |
| - Chemotherapy - ref | | 45.16 | | 41.85 48.48 |  |  |  |
| - Combination/ surgery / radiotherapy | | 45.47 | | 42.66 48.27 | 0.30 | -4.03 4.65 | 0.890 |

*p ≤ 0.10, ** p ≤ 0.05

^1^ *marginal mean and confidence interval*

^2^ *not computed because there were no categories*

^3^ *low (compulsory school) vs. middle (vocational training or high school graduation) vs. high (secondary or university education)*

^4^*Net income of the household in Swiss Francs (CHF)*
